# Drug take-back program: assessment of knowledge, practices, and barriers to safe disposal of unused medication among healthcare students in a Nigerian university

**DOI:** 10.1186/s12909-023-04788-y

**Published:** 2023-10-27

**Authors:** Wuraola Akande-Sholabi, Damilola Q. Olaoye, Yusuff A. Adebisi

**Affiliations:** 1 Department of Clinical Pharmacy and Pharmacy Administration, Faculty of Pharmacy, University of Ibadan, Ibadan, Nigeria; 2 Public Health Initiative, University of Ibadan, Ibadan, Nigeria; 3 Nuffield Department of Population Health, University of Oxford, Oxford, UK

**Keywords:** Unused medicines, Safe disposal, Healthcare students, Take-back program

## Abstract

**Background:**

The safe disposal of unused medication is a critical public health issue, with risks including environmental pollution, accidental ingestion, and misuse. Inadequate adherence to proper disposal methods among healthcare students could affect the practice of safe disposal of unused medicines as future healthcare professionals. This study, conducted at a Nigerian university, aimed to assess the knowledge, adherence to safe disposal practices, and barriers faced by healthcare professional students regarding unused medication disposal.

**Methods:**

A cross-sectional study was carried out among 930 healthcare students in a Nigerian University, comprising medical and surgery, nursing, pharmacy, physiotherapy, and medical laboratory science students. Information was gathered from respondents using a self-administered questionnaire. Multivariate analyses were used to evaluate the relationship between specific variables and participants’ knowledge and practice scores, while chi-square and logistic regression tests were used for categorical variables at *p < 0.05*.

**Results:**

A total of 930 students participated in this study. The results revealed a significant gap in knowledge, with (67.7%; 630) of the participants unaware of proper disposal methods and most scoring either 0 (31.9%; 297) or 1 (46.0%; 428) out of 4 on a knowledge-based questionnaire. Pharmacy students were the most knowledgeable, with 44.4% falling into the high knowledge category. However, their knowledge did not always correspond to correct practices, with only (10.1%; 94) of participants reporting use of recommended disposal methods such as returning unused medicine to a pharmacy or a drug take-back program. Significant associations were found with course of study (χ²=12.14, p = 0.033) and awareness of correct disposal methods (χ²=4.035, p = 0.045). Those aware of the correct disposal method had a higher odds ratio of high knowledge score (OR = 1.62, 95% CI: 1.1–2.41, p = 0.018) compared to those who were not aware. In terms of disposal practices, those who had received training on safe disposal had significantly higher odds of good practice score (OR = 2.25, 95% CI: 1.36–3.74, p = 0.002). Major barriers identified included lack of access to drug take-back programs (50.4%; 469), lack of knowledge (36.8%; 342), and inconvenience (10.3%; 14).

**Conclusion:**

A knowledge gap was revealed among the respondents regarding the safe disposal of unused medications. Despite the presence of knowledge and awareness, these do not necessarily translate into good disposal practices. This call for strategies to overcome identified barriers, with the aim to bridge the knowledge-practice gap and promote safe disposal of unused medication. The study underscores the urgent need for improved public health policies and educational programs on safe medication disposal.

## Introduction

Pharmaceutical products are essential in maintaining human health, despite this advantage, there exist inherent risks associated with the misuse or mismanagement of drugs during or post-usage [1, 2]. The consumption of prescription and over-the-counter medicines is escalating globally and was projected to reach 4.5 trillion doses in 2020, signifying a 24% increase from 2015. Unquestionably, not all prescribed drugs are consumed by the patients, leading to a significant volume of medication either remaining unused or circulating past their expiration date [3]. In recent years, the unsafe disposal of drugs has escalated into a global concern, largely due to a considerable proportion of circulating drugs remaining unused. Reasons for this include, but are not limited to, expiration, excessive prescription practices, home storage, undesirable side effects, manufacturer promotions, and the medication-seeking behaviour of individuals worldwide [4–6]. Households often store medications leading to wastage, typically in anticipation of their use in acute, chronic, or emergency situations [7]. The magnitude of this issue has spurred studies among different population groups, including students, to comprehend and address the problem more effectively.

Several studies in Africa have revealed that many families store drugs at home. In Sudan, nearly all investigated households had at least one drug product [8]. In Uganda, 40% of the surveyed homes contained medicines, and 30% had antibacterial reserved for future use [9]. A study conducted about 2 decades ago in Addis Ababa, Ethiopia, discovered that 20% of the homes had stored drugs, and 17% of the residents were sharing them [10]. The accumulation of drugs in these homes frequently leads to leftover and expired drugs. The improper disposal of unused medicines can pose significant environmental hazards and health threats, such as acute poisoning, danger to aquatic life, and accidental use, particularly in children, in addition to contributing to antimicrobial resistance [11]. Increasing evidence from research supports that the build-up of antibiotics in our waterways can exacerbate antibiotic resistance and alter the virulence phenotypes of microorganisms [12]. The contamination of water bodies by birth control pills has reportedly caused endocrine disruption, impairing the sexual development of roaches and fish [13, 14]. Reports from Pakistan have associated exposure to diclofenac residues with a substantial decline in the population of Oriental white-backed vultures [15]. Moreover, even trace amounts of organic contaminants from pharmaceuticals have been detected in drinking water treatment plants [16].

In Nigeria, the issues of drug wastage and improper drug disposal have not been extensively studied. However, the National Agency for Food and Drug Administration and Control (NAFDAC) has established standard guidelines for the disposal of expired drugs [17]. A study in Nigeria reported that only about 23% of the respondents, who were community pharmacists, complied with these standard guidelines for drug disposal [18]. This study, alongside numerous others conducted across various health professions in Africa, emphasizes the need to understand the knowledge, practices, and barriers related to the safe disposal of unused medicine among health professionals. As future prescribers and dispensers of medications, healthcare students have a pivotal role in mitigating the improper disposal of unused medication. Consequently, there has been a surge in research reports focusing on exploring students’ knowledge, practices, and barriers towards the safe disposal of medication [19]. Previous studies conducted among healthcare students have revealed a disparity between knowledge and practice, highlighting the necessity for educational interventions to improve knowledge and awareness [20, 21].

The safe disposal of unused medication is a crucial component of public health and environmental stewardship. It is essential to assess the knowledge, practices, and obstacles faced by healthcare professional students regarding the safe disposal of unused medication, given their future role in healthcare delivery. It’s worth noting that an individual’s perception of an idea can influence their behaviour on an issue, thereby creating behavioural and contextual barriers. Evidence suggests that strategic actions such as drug take-back programs need to be implemented. Therefore, enhancing healthcare students’ knowledge, particularly pharmacy students, is crucial in combating the unsafe disposal of unused drugs. Well-educated pharmacists can contribute significantly to reducing the improper disposal of medications. Moreover, in Nigeria, most previous studies on the inappropriate disposal of unused medication have focused on general populations [22, 23], with limited information on healthcare students.

Therefore, this study is centred on a Nigerian university and aims to conduct an assessment to gain insights into the current state of knowledge, adherence to safe disposal practices, and the barriers faced by healthcare professional students in this regard. By identifying gaps and challenges, this research aims to inform educational interventions, policy development, and resource allocation to enhance safe medication disposal practices among future healthcare professionals in Nigeria, ultimately contributing to improved public health outcomes and environmental sustainability.

## Method

### Study design settings

This cross-sectional study was conducted at a university and employed an online questionnaire, which participants completed independently. The study encompassed undergraduate students from various departments, including Pharmacy, Nursing, Physiotherapy, Medical Laboratory Science, and Medicine and Surgery. The class representative of each class was identified, then the google link was shared with the class representative who thereafter distributed the questionnaire via the students’ class WhatsApp group. Data collection occurred between May and June 2023. In Nigeria, the Bachelor of Pharmacy, Bachelor of Physiotherapy, Bachelor of Medical Laboratory Science, and Bachelor of Nursing programs typically span five years, while the Bachelor of Medicine and Surgery program lasts for six years. The study’s eligible participants were registered undergraduate students from the aforementioned departments who willingly agreed to participate. Students who did not provide consent were excluded from the study.

### Sample size determination

Based on the information provided by the faculty management, there were a total of 1865 registered students across various departments: Pharmacy (387), Medicine and Surgery (898), Nursing (220), Physiotherapy (170), and Medical Laboratory Sciences (190), this served as sample population. The study aimed to achieve a 95% confidence level and a 5% alpha error. Utilizing Yamane’s formula [24], the calculated sample size was determined to be 316. When applying this formula to each specific department, the target sample sizes were proportionally distributed as follows: Pharmacy (65), Medicine and Surgery (150), Physiotherapy (32), Medical Laboratory Science (35), and Nursing (37). Additionally, to account for the possibility of a low response rate, which is common among students, an attrition rate of approximately 10% was taken into consideration. This adjustment aimed to ensure that the sample size remained statistically valid even if there was a significant lack of participation.

### Sampling and data collection procedure

The sampling and data collection procedure entailed the dissemination of online questionnaire links within students’ class groups. The links to online questionnaires were shared among the student groups, with consent already sought prior to dissemination. The respondents provided voluntary informed consent by filling the online questionnaire. Participation in the study was strictly voluntary, and students were informed of their right to withdraw their participation at any time. All respondents were guaranteed anonymity and confidentiality. To ensure the integrity of the data, measures were instituted to prevent multiple questionnaire submissions by the same respondents. For instance, the Google Form was set to allow only one response per Google account, ensuring that respondents could not easily submit multiple entries.

### Data collection instrument

Data was collected using a pre-tested, self-administered questionnaire derived from previous studies conducted by Ayele and Mamu [25]. The questionnaire was slightly modified by the investigators, incorporating the FDA guide on the safe disposal of unused medicines to align with our specific setting. The questionnaire comprised four sections as follows:

Section 1: This section captured the participants’ demographic characteristics. Section 2: Comprised of four questions assessing participants’ knowledge regarding the safe disposal of unused medicine. Section 3: Included four questions examining participants’ practices concerning the safe disposal of unused medicines. Section 4: Contained two questions evaluating participants’ perceived barriers to the safe disposal of unused medicines.

The responses to practice and knowledge-related questions were scored with each correct response earning one point. The possible practice score ranged from 0 to 4, while the possible knowledge score ranged from 0 to 4. These scores were then categorized into ‘good’ or ‘poor’ for practice, and ‘high’ or ‘low’ for knowledge. A practice score of 2, 3, or 4 was considered ‘good’ practice, while a score of 0 or 1 indicated ‘poor’ practice. Similarly, a knowledge score of 2, 3, or 4 was categorized as ‘high’ knowledge, while a score of 0 or 1 represented ‘low’ knowledge.

### Pretest and content validation

The questionnaire’s content validity was confirmed by a multidisciplinary team, including a nurse, a pharmacist, a physiotherapist, and a medical doctor engaged in academic work. This process aimed to ensure that the questionnaire appropriately reflected the study’s specific objectives and justifications. Face validity was established through a pretest involving forty students from the Pharmacy, Physiotherapy, Nursing, Medical Laboratory Science, and Medicine and Surgery departments. This pertest aimed to evaluate students could understand and respond to the questionnaire’s various questions. The feedback from this process was then used to make further modifications to the research instrument, optimizing it for the intended respondents. The students involved in the validity testing were not included in the actual study to avoid any potential bias or influence on the survey’s overall results. Their contribution was solely for refining the questionnaire and ensuring its appropriateness and comprehensibility for the broader student population participating in the study.

### Statistical analysis

Initial data cleaning and pre-processing were performed using Microsoft Excel. The cleaned data were then exported to Stata 17 for detailed statistical analysis. Descriptive statistics were used to summarize participants’ demographics and questionnaire responses. Associations between demographic characteristics and the level of knowledge and practice on safe medication disposal were analysed using Chi-square tests. Logistic regression analysis was employed to explore relationships between significant variables and knowledge of safe medication disposal, with a level of significance set at p < 0.05. Descriptive statistics was used to report the barriers and the suggestion by the participants to curb unsafe disposal of unused medicine.

### Ethics approval

Ethical clearance for this study was granted by the Joint University Institutional Review Board with approval number UI/EC/23/0351.

## Results

Notably, 83.8% (n = 779) reported having no training on safe disposal of medications, and 67.7% (n = 630) were unaware of proper disposal methods, indicating prevalent gaps in both training and awareness. Detailed demographic data is presented in Table 1.

**Table 1:** Demographic Characteristics of the Study Participants (n = 930)

Demographic Characteristics	Category	Frequency (n)	Percentage (%)
**Gender**	Male	472	50.8
	Female	458	49.2
**Age group**	18–20	231	24.8
	21–25	586	63.0
	26–30	99	10.7
	> 30	14	1.5
**Level of Study**	100	191	20.5
	200	294	31.6
	300	183	19.7
	400	105	11.3
	500	114	12.3
	600	43	4.6
**Course of Study**	Medicine and Surgery	292	31.4
	Pharmacy	342	36.8
	Nursing	100	10.8
	Medical Lab Science	91	9.8
	Physiotherapy	89	9.6
	Others	16	1.7
**Place of Residence**	University-owned	626	67.3
	Private Accommodation	304	32.7
**Received Training on Safe Disposal**	Yes	151	16.2
	No	779	83.8
**Aware of Correct Disposal Method**	Yes	300	32.3
	No	630	67.7

In the knowledge-based questionnaire (Table 2), a large proportion of participants demonstrated misconceptions in subsequent questions, with only 8.3% (n = 77) and 21.1% (n = 196) correctly answering questions 2 and 3, respectively. The overall knowledge scores demonstrated a low level of understanding among participants. The majority scored either 0 (31.9%, n = 297) or 1 (46.0%, n = 428) out of 4, while only 22.0% (n = 205) were classified as having a high knowledge score (2, 3, or 4). These results indicate a widespread lack of comprehensive knowledge about safe medicine disposal methods.

**Table 2:** Responses to the knowledge-related questions and knowledge score of the study participants (n = 930)

Knowledge-related questions	Frequency (n)	Percentage (%)
**Q1. Which of the following is the safest way to dispose of unused medicine?**		
Flush it down the toilet	117	12.6
Put it in the trash	220	23.7
Return it to a pharmacy or drug take-back program (Correct Answer)	559	60.1
Give it to someone who needs it	34	3.7
**Q2. Please select the reasons you believe the methods mentioned in question 1 are safe or unsafe. (Choose all that apply)**		
Potential for environmental contamination	583	62.7
Risk of accidental ingestion by children or pets	716	77.0
Possibility of drug abuse or misuse	618	66.5
Legal or regulatory concerns	427	45.9
Accessibility to proper disposal options	485	52.2
Choose all of the above (Correct Answer)	77	8.3
**Q3. In the absence of a take-back program, how should solid medicines be disposed of?**		
Mix them with an unpalatable substance (e.g., coffee grounds or cat litter) and place in a sealed container before disposing in the trash (Correct Answer)	196	21.1
Crush the solid medicines and flush them down the toilet	420	45.2
Place them directly in the trash without any modifications	194	20.9
Donate them to a local charity or medical facility	120	12.9
**Q4. How do you think improper disposal of unused medication can affect the environment and public health? (Choose all that apply)**		
Contamination of water supplies and aquatic ecosystems	772	83.0
Accumulation of pharmaceutical residues in the soil	691	74.3
Development of antibiotic-resistant bacteria	579	62.3
Increased risk of accidental ingestion by children, pets, or wildlife	777	83.5
Facilitation of drug abuse and misuse		
All of the above (Correct Answer)	20	2.15
**Knowledge score**		
0	297	31.9
1	428	46.0
2	191	20.5
3	14	1.5
4	0	0
**Knowledge score category**		
Low Knowledge (Score 0, or 1)	725	78.0
High Knowledge (Score 2, 3 or 4)	205	22.0

The association between the knowledge score category and demographic characteristics of the participants is outlined in Table 3. Chi-square tests were used to analyze these associations. Regarding gender, there was no significant association between gender (χ²=2.00, p = 0.156), age group (χ²=0.73, p = 0.867) or level of study (χ²=8.77, p = 0.119) and knowledge score category. In contrast, the course of study showed a significant association with the knowledge score category (χ²=12.14, p = 0.033). Pharmacy students were notably more represented in the high knowledge category (44.4%), while the representation of Medical Laboratory Science students was lower in the high knowledge category (5.9%). Furthermore, a significant association was found between the awareness of correct disposal methods and the knowledge score category (χ²=4.035, p = 0.045). Participants with high knowledge were more likely to be aware of the correct disposal method (38.1%) compared to those with low knowledge (30.6%).

**Table 3:** Association between knowledge score category and demographic characteristics (n = 930)

Demographic characteristics	Low Knowledge (n = 725)	High Knowledge (n = 205)	Chi-square (p-value)
**Gender**			χ²(df = 1) = 2.00, p = 0.156
Male	359 (49.5)	113 (55.1)	
Female	366 (50.5)	92 (44.9)	
**Age group, years, n (%)**			χ²(df = 3) = 0.73, p = 0.867
18–20	178 (24.6)	53 (25.9)	
21–25	459 (63.3)	127 (62.0)	
26–30	76 (10.5)	23 (11.2)	
> 30	12 (1.7)	2 (0.98)	
**Level of Study, n (%)**			χ²(df = 5) = 8.77, p = 0.119
100	144 (19.9)	47 (22.9)	
200	236 (32.6)	58 (28.3)	
300	150 (20.7)	33 (16.1)	
400	83 (11.5)	22 (10.7)	
500	84 (11.6)	30 (14.6)	
600	28 (3.9)	15 (7.3)	
**Course of Study, n (%)**			χ²(df = 5) = 12.14, p = 0.033
Medicine and Surgery	227 (31.3)	65 (31.7)	
Pharmacy	251 (34.6)	91 (44.4)	
Nursing	83 (11.5)	17 (8.3)	
Medical Laboratory Science	79 (10.9)	12 (5.9)	
Physiotherapy	70 (9.7)	19 (9.3)	
Others	15 (2.1)	1 (0.5)	
**Place of residence, n (%)**			χ²(df = 1) = 0.113, p = 0.737
Private accommodation	235 (32.4)	69 (33.7)	
University owned	490 (67.6)	136 (66.3)	
**Received Training on Safe Disposal, n (%)**			χ²(df = 1) = 0.076, p = 0.783
Yes	119 (16.4)	32 (15.6)	
No	606 (83.6)	173 (84.4)	
**Aware of Correct Disposal Method, n (%)**			χ²(df = 1) = 4.035, p = 0.045
Yes	222 (30.6)	78 (38.1)	
No	503 (69.4)	127 (61.9)	

Table 4 presents the results of the logistic regression analyses for the knowledge score category, adjusted for demographic characteristics. When the course of study was adjusted for demographic characteristics, the Pharmacy course was used as the reference group. The Medical Laboratory Science course had a significantly lower odds ratio of 0.40 (95% CI: 0.21–0.78, p = 0.01), indicating that students in this course were less likely to fall into the high knowledge category compared to Pharmacy students. Other courses of study such as Medicine and Surgery, Nursing, and Physiotherapy did not show a statistically significant difference compared to the reference group (p-values: 0.08, 0.12, and 0.37, respectively). In terms of awareness of the correct disposal method, when adjusted for demographic characteristics, those who were aware of the correct disposal method had an odds ratio of 1.62 (95% CI: 1.1–2.41, p = 0.018), indicating that these individuals were significantly more likely to fall into the high knowledge category compared to those who were not aware of the correct disposal method.

**Table 4:** Multivariate Logistic Regression Results for Knowledge Score Category Adjusted for Demographic Characteristics (n = 930)

Demographic characteristics	Odds Ratio (95% CI)	p-value
Course of Study*		
Medicine and Surgery	0.69 (0.46–1.04)	0.08
Pharmacy	Reference group	
Nursing	0.62 (0.34–1.14)	0.12
Medical Laboratory Science	0.40 (0.21–0.78)	0.01
Physiotherapy	0.75 (0.41–1.39)	0.37
Aware of Correct Disposal Method**		
Yes	1.62 (1.10–2.41)	0.018
No	Reference group	

*Adjusted for gender, age group, level of study place of residence, past training on safe disposal and awareness of correct disposal of medicine

**Adjusted for gender, age group, level of study place of residence, past training on safe disposal and course of study

Table 5 displays the responses to practice-related questions and their resulting practice scores among study participants. The most common method of unused medicine disposal was throwing it in the trash (67.1%). Only a small proportion of participants (10.1%) reported using recommended methods such as returning unused medicine to a pharmacy or a drug take-back program. A significant portion of the study participants (46.2%) reported previously disposing of unused medication in a manner they later discovered was unsafe. Looking at the practice scores, most participants exhibited poor practice scores with 26.2% scoring 0 and 48.6% scoring 1. Only a quarter (25.2%) fell into the good practice category, represented by scores of 2, 3, or 4. This indicates a predominance of poor practices regarding the disposal of unused medication among the study population.

**Table 5:** Responses to the practice-related questions among study participants (n = 930)

Practice-related questions	Frequency (n)	Percentage (%)
What do you usually do with unused medicine?		
Throw it in the trash	624	67.1
Flush it down the toilet	88	9.5
Return it to a pharmacy or drug take-back program	94	10.1
Give it to someone who needs it	124	13.3
**Have you ever disposed of unused medication in a way that you later found out was unsafe?**		
Yes	430	46.2
No	500	53.8
**Do you keep unused medication at home?**		
Yes	690	74.2
No	240	25.8
**Have you ever advised anyone on how to safely dispose of unused medication?**		
Yes	144	15.5
No	786	84.5
**Practice Score**		
0	244	26.2
1	452	48.6
2	182	19.6
3	46	5.0
4	6	0.7
**Practice score category**		
Good practice (Score 2, 3 or 4)	234	25.2
Poor practice (Score 0, or 1)	696	74.8

Table 6 presents the association between the practice score category and demographic characteristics of the study participants. Notably, there were significant differences in the practice score categories when stratified by gender (p = 0.006), age group (p < 0.001), training on safe disposal (p < 0.001), and awareness of the correct disposal method (p < 0.001). More males (58.5%) were in the good practice category compared to females (41.5%). Among age groups, the highest proportion of good practice was observed in the 21–25 years group (59.8%). However, the good practice increased with age, being the lowest in the 18–20 years group (20.1%) and highest in the > 30 years group (3.0%). Participants who had received training on safe disposal were more likely to exhibit good practices (27.4%) than those who had not received such training (12.5%). Similarly, awareness of the correct disposal method was also associated with good practice. Participants who were aware of the correct disposal method had a higher proportion of good practice (44.0%) compared to those who were not aware (28.3%). No significant differences in practice score categories were found when stratified by level of study (p = 0.073), course of study (p = 0.145), and place of residence (p = 0.468).

**Table 6:** Association between practice score category and demographic characteristics (n = 930)

Demographic characteristics	Good Practice (n = 234)	Poor Practice (n = 696)	Chi-square (p-value)
**Gender**			χ²(df = 1) = 7.19, p = 0.0073
Male	137 (58.5)	335 (48.1)	
Female	97 (41.5)	361 (51.9)	
**Age group**			χ²(df = 3) = 20.1, p < 0.001
18–20	47 (20.1)	184 (26.4)	
21–25	140 (59.8)	446 (64.1)	
26–30	40 (17.1)	59 (8.5)	
> 30	7 (3.0)	7 (1.0)	
**Level of Study**			χ²(df = 5) = 10.01, p = 0.073
100	53 (22.7)	138 (19.8)	
200	73 (31.2)	221 (31.8)	
300	39 (16.7)	144 (20.7)	
400	21 (9.0)	84 (12.1)	
500	30 (12.8)	84 (12.1)	
600	18 (7.7)	25 (3.6)	
**Course of Study**			χ²(df = 5) = 8.21, p = 0.145
Medicine and Surgery	58 (24.8)	234 (33.6)	
Pharmacy	88 (37.6)	254 (36.5)	
Nursing	31 (13.3)	69 (9.9)	
Medical Laboratory Science	28 (12.0)	63 (9.1)	
Physiotherapy	24 (10.3)	65 (9.3)	
Others	5 (2.1)	11 (1.6)	
**Place of residence**			χ²(df = 1) = 0.5183, p = 0.42
Private accommodation	81 (34.6)	223 (32.0)	
University owned	153 (65.4)	473 (68.0)	
**Received Training on Safe Disposal**			χ²(df = 1) = 27.32, p < 0.001
Yes	64 (27.4)	87 (12.5)	
No	170 (72.7)	609 (87.5)	
**Aware of Correct Disposal Method**			χ²(df = 1) = 19.785, p < 0.001
Yes	103 (44.0)	197 (28.3)	
No	131 (56.0)	499 (71.7)	

Table 7 provides the results of logistic regression for the practice score category, adjusted for demographic characteristics. After adjustment, being male was significantly associated with higher odds of having a good practice score compared to females (OR = 1.65, 95% CI: 1.18–2.32, p = 0.004). Regarding age, compared to the reference group (18–20 years), the odds of good practice score increased significantly with age. Those who had received training on safe disposal had significantly higher odds of a good practice score compared to those who had not (OR = 2.25, 95% CI: 1.36–3.74, p = 0.002). However, being aware of the correct disposal method was not significantly associated with the practice score category (OR = 1.20, 95% CI: 0.80–1.80, p = 0.371).

**Table 7:** Logistic Regression Results for Practice Score Category Adjusted for Demographic Characteristics (n = 930)

Demographic characteristics	Odds Ratio (95% CI)	p-value
Gender*		
Male	1.65 (1.18–2.32)	0.004
Female	Reference group	
Age group**		
18–20	Reference group	
21–25	1.35 (0.89–2.07)	0.162
26–30	2.25 (1.19–4.28)	0.013
> 30	4.42 (1.38–14.2)	0.012
Received Training on Safe Disposal***		
Yes	2.25 (1.36–3.74)	0.002
No	Reference group	
Aware of Correct Disposal Method****		
Yes	1.20 (0.80–1.80)	0.371
No	Reference group	

*Adjusted for age group, level of study, place of residence, course of study past training on safe disposal and awareness of correct disposal of medicine

**Adjusted for gender, level of study, place of residence, awareness of correct disposal of medicine, past training on safe disposal and course of study

***Adjusted for gender, age group, level of study, place of residence, awareness of correct disposal of medicine, and course of study

****Adjusted for gender, age group, level of study, place of residence, past training on safe disposal and course of study

The three major barriers reported by the participants regarding safe disposal of unused medicine are lack of access to drug take-back programs (50.4%, n = 469), lack of knowledge (36.8%, n = 342), and inconvenience (10.3%, n = 14). Fear of legal repercussions was reported as a barrier by a small proportion of participants (1.5%, n = 96). The participants suggested strategies to overcome these barriers include increasing public awareness and education on safe disposal practices (94.6%, n = 880), providing more accessible drug take-back programs (78.5%, n = 730), and encouraging healthcare providers to discuss proper disposal with patients (75.7%, n = 704). Additional suggestions include developing and implementing policies that encourage proper medication disposal (70.2%, n = 653) and increasing penalties for improper disposal (31.2%, n = 290).

## Discussion

The findings from this study raise important implications for public health policies regarding the safe disposal of medicines. It appears that a large portion of university students, in this case, were not adequately trained or aware of the correct methods for disposing of medications. This is a significant concern considering that previous research has shown that improper disposal of medications can lead to various environmental and health risks [5–7]. Similar to this study, previous studies also highlighted that a large proportion of healthcare students lacked knowledge about correct medicine disposal methods and stressed the importance of implementing more effective training programs [26–28].

Interestingly, while the knowledge scores in this study were generally low, it was observed that pharmacy students had significantly higher knowledge scores. This is in line with the findings of Shakib et al. [29], which demonstrated that healthcare students, particularly pharmacy students, generally have a better understanding of safe disposal practices. This suggests that targeted education programs could help bridge the knowledge gap in non-pharmacy students. Moreover, this study revealed that gender, age, training on safe disposal, and awareness of correct disposal methods significantly influenced the practice scores for safe disposal of medications. Hence, the study strongly advocates for targeted educational interventions to enhance understanding and practices for safe medication disposal among the university students, which would effectively reduce the risk of improper disposal and its associated hazards. The link between training and better disposal practices observed in this study echoes the findings of Jarvis et al. [30], reinforcing the importance of proper education and awareness programs in ensuring the safe disposal of unused medicines.

However, it is notable that the association between knowledge and good practice was not statistically significant, suggesting that knowledge alone might not be enough to guarantee safe disposal practices. This could be due to various barriers like lack of access to drug take-back programs and inconvenience, as identified in this study. Previous studies have also identified similar barriers and emphasized the need for policies to make drug take-back programs more accessible and convenient [31–33].

This study’s strengths include a substantial sample size, which enhances the reliability and generalizability of the findings within the university student population. The study also provides a comprehensive analysis of the knowledge and practices of medicine disposal, providing unique insights into the factors that influence safe disposal behaviours. Furthermore, it successfully identifies barriers to safe medicine disposal and proposes tangible solutions, contributing valuable recommendations for improving public health policies and education programs. However, this study is not without limitations. Firstly, the cross-sectional design may not capture changes in knowledge and practices over time. Secondly, participants were only selected from one institution, limiting the generalizability of findings. Additionally, the self-reported nature of data may have introduced social desirability bias, affecting the accuracy of responses. Future studies should utilize a longitudinal design and larger, more diverse samples to capture a more comprehensive view of medication disposal practices. Interventions targeting training and awareness should be designed, and their impact on improving safe disposal practices should be evaluated. Policies encouraging proper medication disposal should also be developed and implemented.

## Conclusions

This study reveals a significant knowledge gap among healthcare professional university students regarding the safe disposal of unused medications, with only a minority demonstrating high knowledge and good practice scores. Pharmacy students were an exception, reflecting better understanding and adherence to proper disposal methods. Notably, the presence of training, awareness of appropriate disposal methods, and demographic factors like age and gender significantly impacted practice scores. However, knowledge alone did not guarantee good disposal practices, highlighting the need to address barriers such as lack of access to drug take-back programs and inconvenience. Therefore, improving public health policies and education programs on the safe disposal of medicines is crucial, making these more comprehensive, accessible, and efficient.

## Data Availability

The datasets used and/or analysed during the current study are available from the corresponding author on reasonable request.

## Abbreviations

NAFDAC: National Agency for Food and Drug Administration and Control
